# High-resolution diffusion imaging in the unfixed post-mortem infant brain at 7 T

**DOI:** 10.1162/imag_a_00069

**Published:** 2024-01-22

**Authors:** Wenchuan Wu, Sebastian W. Rieger, Luke Baxter, Eleri Adams, Jesper L.R. Andersson, Maria M. Cobo, Foteini Andritsou, Matteo Bastiani, Ria Evans Fry, Robert Frost, Sean Fitzgibbon, Sean Foxley, Darren Fowler, Chris Gallagher, Amy F.D. Howard, Joseph V. Hajnal, Fiona Moultrie, Vaneesha Monk, David Andrew Porter, Daniel Papp, Anthony Price, Jerome Sallet, Michael Sanders, Dominic Wilkinson, Rebeccah Slater, Karla L. Miller

**Affiliations:** Wellcome Centre for Integrative Neuroimaging, FMRIB, Nuffield Department of Clinical Neurosciences, University of Oxford, Oxford, United Kingdom; Oxford Centre for Human Brain Activity, Wellcome Centre for Integrative Neuroimaging, Department of Psychiatry, University of Oxford, Oxford, United Kingdom; Department of Paediatrics, University of Oxford, Oxford, United Kingdom; Newborn Care Unit, John Radcliffe Hospital, Oxford University Hospitals NHS Foundation Trust, Oxford, United Kingdom; Universidad San Francisco de Quito USFQ, Colegio de Ciencias Biologicas y Ambientales, Quito, Ecuador; Sir Peter Mansfield Imaging Centre, School of Medicine, University of Nottingham, Nottingham, United Kingdom; NIHR Biomedical Research Centre, University of Nottingham, Nottingham, United Kingdom; Athinoula A. Martinos Center for Biomedical Imaging, Massachusetts General Hospital, Charlestown, MA, United States; Department of Radiology, Harvard Medical School, Boston, MA, United States; Department of Radiology, University of Chicago, Chicago, IL, United States; Centre for the Developing Brain, School of Biomedical Engineering and Imaging Sciences, King’s College London, King’s Health Partners, St. Thomas’ Hospital, London, United Kingdom; Department of Biomedical Engineering, School of Biomedical Engineering and Imaging Sciences, King’s College London, King’s Health Partners, St. Thomas’ Hospital, London, United Kingdom; Imaging Centre of Excellence, College of Medical, Veterinary & Life Sciences, University of Glasgow, Glasgow, United Kingdom; Department of Experimental Psychology, University of Oxford, Oxford, United Kingdom; Univ Lyon, Université Lyon 1, Inserm, Stem Cell and Brain Research Institute U1208, Bron, France; Oxford Uehiro Centre for Practical Ethics, Oxford, United Kingdom; John Radcliffe Hospital, Oxford, United Kingdom

**Keywords:** diffusion imaging, neonate, post-mortem MRI, 7 T, high resolution, unfixed

## Abstract

Diffusion MRI of the infant brain allows investigation of the organizational structure of maturing fibers during brain development. Post-mortem imaging has the potential to achieve high resolution by using long scan times, enabling precise assessment of small structures. Technical development for post-mortem diffusion MRI has primarily focused on scanning of fixed tissue, which is robust to effects like temperature drift that can cause unfixed tissue to degrade. The ability to scan unfixed tissue in the intact body would enable post-mortem studies without organ donation, but poses new technical challenges. This paper describes our approach to scan setup, protocol optimization, and tissue protection in the context of the Developing Human Connectome Project (dHCP) of neonates. A major consideration was the need to preserve the integrity of unfixed tissue during scanning in light of energy deposition at ultra-high magnetic field strength. We present results from one of the first two subjects recruited to the study, who died on postnatal day 46 at 29+6 weeks postmenstrual age, demonstrating high-quality diffusion MRI data. We find altered diffusion properties consistent with post-mortem changes reported previously. Preliminary voxel-wise and tractography analyses are presented with comparison to age-matched in vivo dHCP data. These results show that high-quality, high-resolution post-mortem data of unfixed tissue can be acquired to explore the developing human brain.

## 1 Introduction

Post-mortem brain imaging allows acquisition of higher spatial resolution data with improved tissue contrast compared to in vivo image acquisition, facilitating the link between MRI and gold-standard histological findings (Roebroeck et al., 2019). Post-mortem images are also not contaminated with head motion and physiological signal artifacts, which degrade MRI data and derived metrics in vivo (Bastiani et al., 2019; Yendiki et al., 2014).

However, post-mortem MRI also presents unique and complex challenges and limitations. Most previous post-mortem MRI studies image fixed tissue (Huang et al., 2009; McNab et al., 2009; Miller et al., 2011; Pfefferbaum et al., 2004), which is robust to factors such as temperature increases that can cause rapid degradation of unfixed tissue. However, scanning fixed tissue requires brain donation, which can be objectionable to potential donors for a range of reasons. In comparison, the ability to scan unfixed tissue in the intact body allows post-mortem imaging without brain donation, creating more acceptable conditions for study participation. In addition, tissue fixation poses technical challenges to imaging because it dramatically reduces T2 relaxation times and diffusivities (Pfefferbaum et al., 2004; Shepherd et al., 2009); this negatively impacts signal-to-noise ratio (SNR), requiring acquisitions lasting many hours. Imaging of unfixed tissue with higher T2 and diffusivity has potential to achieve high resolution in shorter scan durations, particularly at ultra-high magnetic field strength (>3 T). It is worth noting that, in unfixed tissues, diffusivity would also be reduced compared to in vivo because the brain is ischemic and the tissue temperature is lower (e.g., at room temperature). In this study, we aim to acquire high-resolution diffusion MRI data from unfixed post-mortem infant brain at 7 T, as part of the Developing Human Connectome Project (dHCP) (http://www.developingconnectome.org).

Previous post-mortem MRI studies of unfixed human fetuses at ultra-high field (Scola et al., 2018; Thayyil et al., 2009; Votino et al., 2012) acquired short (<2 hours) structural scans as an alternative to autopsy, demonstrating improved resolution and diagnostic accuracy over conventional MRI. Diffusion MRI of unfixed adult (Scheurer et al., 2011) and neonate brains (McDowell et al., 2018; Papadopoulou et al., 2016; Scola et al., 2018) have also been reported at 1.5 T and 3 T with a relatively low spatial resolution and short scan time (<30 minutes). To our best knowledge, no previous studies have reported high spatial- (i.e., sub-millimeter isotropic) and angular-resolution dMRI data from unfixed neonate brains with an extended scan time. Imaging of unfixed post-mortem tissue faces specific challenges. A key challenge is that imaging unfixed tissue over longer scan times risks tissue damage due to deposited RF energy. Avoiding tissue damage would thus require temperature maintenance, ideally at mortuary temperatures (5-10 °C) to minimize tissue degradation. This challenge is exacerbated at ultra-high field due to higher energy deposition during imaging.

In this paper, we present our approach to acquire high-resolution diffusion MRI (dMRI) data from unfixed post-mortem infant brain at 7 T. A particular goal of our study is to achieve compatibility with the in vivo dHCP dataset (see Section 2.1) (Hutter et al., 2018) while optimizing acquisition parameters for image quality. Optimization of imaging protocols for unfixed post-mortem infant brains at ultra-high field is currently an open challenge due to the lack of knowledge of tissue parameters, which generally differ in infant versus adult brain and post-mortem versus in vivo tissue. Here, we detail the scanning procedure we developed to enable high-resolution data acquisition in a single overnight session shortly after death. The brain temperature was kept at ~10 °C during the dMRI scan using a temperature-controlled cooling system. High b values of 3000, 6000, and 9000 s/mm^2^ were used in this study to improve diffusion contrast in the presence of reduced diffusivities due to post-mortem tissue changes and lower temperature. We present results from one of first two subjects recruited, who died on postnatal day 46 at 29+6 weeks postmenstrual age and was scanned within 18 hours of death. We demonstrate that ultra-high field scanning can provide high-resolution post-mortem dMRI data with sufficient SNR to support robust dMRI modelling. This continuing study aims to facilitate the detailed microstructural assessment of small and intricate brain structures, characterize their developmental trajectories, and track the development of normal and pathological tissue.

## 2 Methods

### 2.1 Diffusion sequence and imaging protocols

Post-mortem dMRI provides both unique opportunities (e.g., longer scan times) and challenges (e.g., problematically altered MR-relevant tissue properties). Post-mortem dMRI studies often leverage long scan times to address tissue changes by employing less conventional diffusion-sensitizing pulse sequences that provide higher signal in tissues with short T2 (Fritz et al., 2019; McNab et al., 2009; Miller et al., 2012). A downside of these approaches is that these diffusion-weighted signals use different signal-forming mechanisms that are not directly comparable with conventional single-shot diffusion-weighted spin-echo. To achieve compatibility with the in vivo dHCP data, we use a diffusion-weighted spin-echo sequence with a readout-segmented acquisition to achieve high spatial resolution without problematic distortion (Holdsworth et al., 2008; Porter & Heidemann, 2009). In addition, we used a simultaneous multi-slice acquisition (Larkman et al., 2001; Setsompop et al., 2013) to mitigate the increased scan time required for readout segmentation (Frost et al., 2015). In this section, we overview the process of sequence and protocol optimization with justifications for key decisions made.

A further challenge is that we did not judge it to be ethical to enroll into our study solely for purposes of piloting. We thus aimed to perform signal predictions to optimize our imaging protocol in advance of our first recruitment to maximize the chance of acquiring usable data. These optimizations faced the additional challenge of incomplete knowledge of the MR-relevant properties of unfixed infant brain at 7 T.

One of our primary goals of this study is to achieve a significantly higher spatial resolution over the main in vivo dHCP protocol. To realize this in the protocol optimization, we started with a target resolution of 0.8 mm isotropic, which is much higher than the 1.5 mm resolution of in vivo dHCP protocol, and optimized the acceleration factors. From there, we worked back to calculate whether the resolution can be further increased.

#### 2.1.1 Q-space sampling

The in vivo dHCP study acquires data at b = 400, 1000, and 2600 s/mm^2^ (Bastiani et al., 2019). However, post-mortem dMRI requires higher b values due to the reduction of diffusivities as a result of lower tissue temperature and ischemic changes after death (Mintorovitch et al., 1991; Sotak, 2002). Previous studies predict diffusivities to be ~5-fold lower compared to in vivo (McDowell et al., 2018; Papadopoulou et al., 2016). Given the paucity of literature on diffusivities and relaxation times for unfixed neonate tissue at 7 T and our challenges with recruiting subjects for pilot scans, we decided to prepare three sets of diffusion protocols before the first scan. To aim for compatibility with in vivo dHCP data, each protocol had three b values, with maximum b values across the candidate protocols of 4000, 6000, and 9000 s/mm^2^. We used these protocols to rapidly pilot whether the limiting factor in a given protocol is SNR or diffusion contrast (favoring lower or higher b values, respectively). In the lowest b-value protocol, b = 4000 s/mm^2^ would provide reduced diffusion contrast relative to the in vivo dHCP b = 2600 s/mm^2^ data even if only considering temperature-induced reductions in diffusivity, representing the minimum acceptable diffusion contrast. In the highest b-value protocol, b = 9000 s/mm^2^ approaches the maximum b value that is achievable on our scanner (b_max = 10,000 s/mm^2^) but was expected to be limited by low SNR. Other protocol parameters (e.g., TE and TR) were optimized for each protocol in accordance with the b value.

During the first post-mortem dHCP scan, we allocated 1 hour at the beginning of the scan session to acquire diffusion datasets with b = 4000, 6000, and 9000 s/mm^2^. The pilot data were acquired at 0.8 mm resolution as the main diffusion protocol (see Section 2.1.4 for details). To reduce the acquisition and reconstruction time, only one diffusion direction was acquired for each b value (along the readout direction), together with one b = 0 image. We did not acquire averages as the SNR was judged to be sufficiently high for all high-b datasets. From this rapid turnaround pilot scan, we found the averaged diffusion coefficient of brain tissue (with a mask excluding background and CSF) in the first post-mortem dHCP infant was 0.164 µm^2^/ms at b = 4000 s/mm^2^, which is comparable with previous findings (McDowell et al., 2018; Papadopoulou et al., 2016). In the in vivo dHCP protocol, a maximum b value of 2600 s/mm^2^ is used. Given the low diffusivity observed in the pilot scan, we decided to use a maximum b value of 9000 s/mm^2^ for better diffusion contrast. This b value approaches our scanner’s maximum achievable b value of 10,000 s/mm^2^. The two lower b values were chosen to be evenly spaced between 0 and the maximum b value. This is similar to the HCP diffusion MRI protocol (https://www.humanconnectome.org/hcp-protocols-ya-3t-imaging), which allows data fitting with different diffusion models, despite not being optimized for any specific model.

Long scan-time diffusion protocols place a high demand on the gradient system, with each individual scan inducing thermal heating that increases with b-value. If this heating exceeds the capacity of the scanner’s cooling system, the scanner will shut down to avoid damage. To minimize gradient load for our given set of directions and b values, the three b shells were segmented into subsets and interleaved (with four, five, and eight subsets for b = 3000, 6000, and 9000 s/mm^2^, respectively). In the scan, the b = 3000 s/mm^2^ and b = 6000 s/mm^2^ protocols were interleaved with the b = 9000 s/mm^2^ protocols to avoid running gradient coils at high duty-cycle continuously for a long period of time. This led to four sub-groups of b = 3000 s/mm^2^ protocols and five sub-groups of b = 6000 s/mm^2^ protocols, interleaved with eight sub-groups of b = 9000 s/mm^2^ protocols. As a worst scenario check, we also tested the b = 9000 s/mm^2^ protocol in a continuous run of 3 hours on a phantom, which completed without causing gradient overheating.

We aligned our sampling scheme to the in vivo dHCP, with 64, 88, and 128 directions for low, medium, and high b values (Tournier et al., 2020). This scheme aims to achieve a balance of dense angular coverage in a given shell to resolve fiber orientations for robust tractography, while also covering three b shells to allow more advanced modelling of diffusion MRI signal (Jensen et al., 2005; Zhang et al., 2012). In addition to the 280 diffusion-weighted volumes, 21 b = 0 volumes were also acquired (totaling 301 image volumes).

#### 2.1.2 Acceleration and scan time

The primary considerations for acceleration are scan time, dictated by the simultaneous multi-slice acceleration, and image quality, reflecting both slice-wise and in-plane acceleration. We consider the former in this section and the latter in the next section.

In simultaneous multi-slice imaging, multiband (MB) RF pulses are used to excite multiple brain slices simultaneously. A major issue with simultaneous multi-slice excitation is the considerably higher power deposition by the radiofrequency (RF) pulses, leading to sample heating. This effect is usually measured by means of “specific energy absorption rate” (SAR) that is related to the average energy dissipated in the body tissue per unit of mass and time. Our 7 T scanner incorporates two SAR modes: a “burst” mode, which allows the sequence to run at the full SAR limit (100%) continuously for a maximum of ~6 minutes, and a “conservative” mode, which allows the sequence to run at ~80% of the full SAR limit continuously for long periods. Due to our longer scans, the post-mortem dHCP study needs to use the “conservative” mode, leading to a stricter SAR constraint.

For our scanner’s specific implementation of diffusion-weighted spin echo with readout-segmented EPI, we compared different combinations of MB and in-plane phase encoding acceleration factor (MB = 1, 2, 3, 4, and R = 1, 2, 3, 4, respectively). We then explored the achievable parameter space of MB factors to determine the optimal choice by evaluating SAR compatibility and the number of diffusion volumes that can be achieved. Acceleration factors higher than 4 are not compatible with the post-mortem infant head coil and were not considered.

**Fig. 1.**
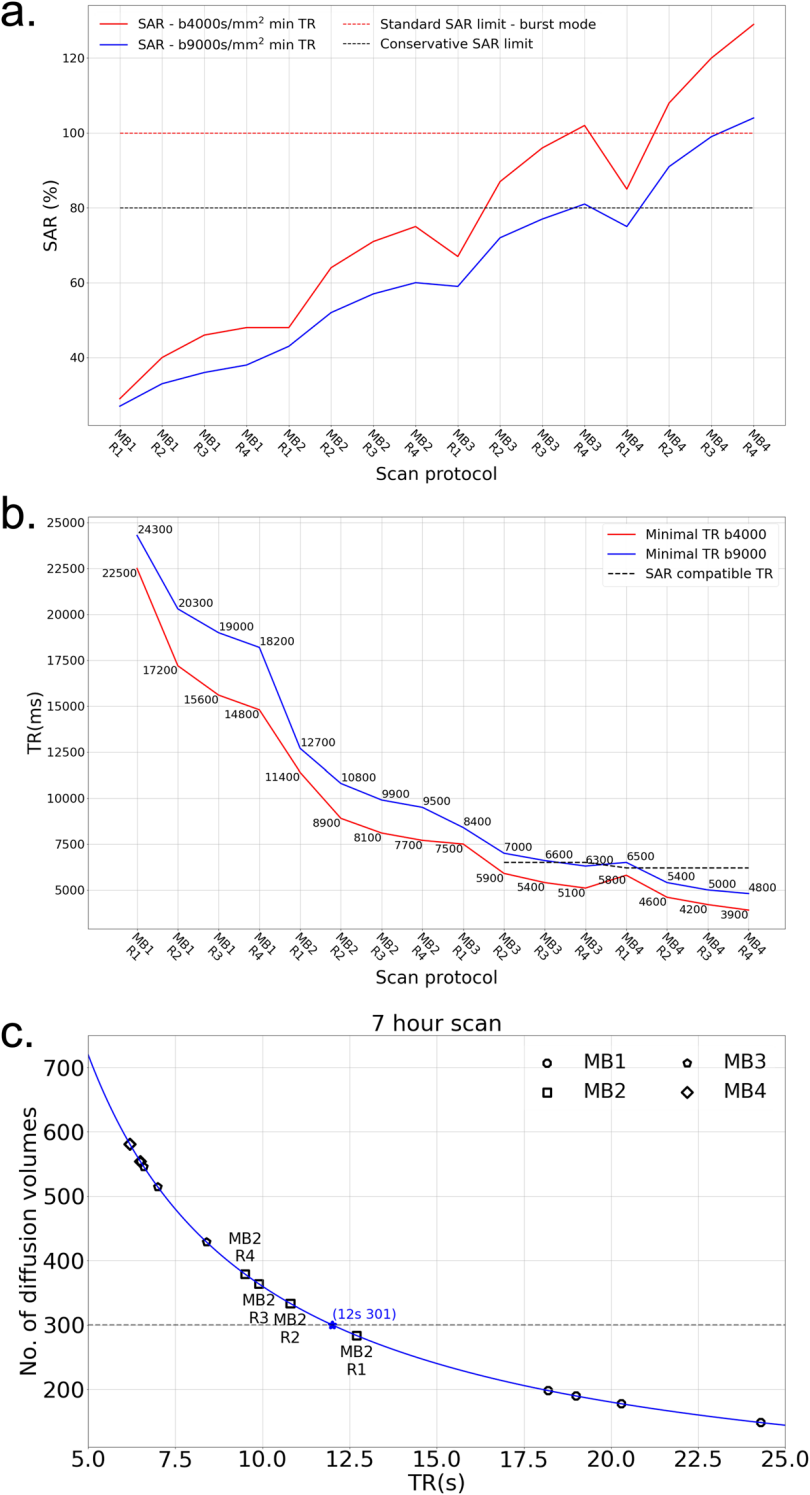
Simulation of accelerations for different protocols. (a) Predicted SAR for different acceleration protocols with minimum allowable TRs. In order to run a protocol consecutively over many hours, the sequence SAR needs to be lower than the conservative SAR limit (black dotted line). Burst mode allows a sequence to run at full SAR limit for a maximum of 6 minutes (red dot line). Protocols with b = 9000 s/mm^2^ (blue solid line) have a lower SAR than the b = 4000 s/mm^2^ protocols (red solid line) due to the long TRs required for large b values. (b) Minimum allowable TRs for all protocols under investigation. To be SAR compatible, some protocols with high MB factors need to use a higher TR (dash line). (c) The relation between the achievable number of diffusion volumes with TRs. In order to acquire at least 301 image volumes in the 7-hour scan, the TR should not exceed 12 s (dash line), which excludes five protocols including all MB = 1 protocols and MB2R1 (i.e., MB factor 2 with in-plane phase encoding acceleration R = 1) protocol.

A first consideration is whether the scanner will impose SAR restrictions for a given protocol, which would disallow the protocol from being run; this is a separate consideration from the tissue heating effects discussed at length above. Figure 1a shows the SAR predicted by the scanner for different acceleration protocols with minimum allowable TRs. High MB factors are associated with higher SARs. Protocols with MB = 3 and 4 for b = 4000 s/mm^2^ and minimum allowable TRs exceed the conservative SAR limit. At b = 9000 s/mm^2^ the requirement for longer TR to accommodate longer diffusion encoding gradients means that MB < 4 protocols are generally not SAR limited. To enable higher acceleration factors, one approach is to increase the TR beyond the minimum. Figure 1b shows minimum allowable TRs for all protocols, where an increase in TR is needed (dashed line) in some protocols with MB = 3 and 4 to be SAR compatible.

Protocols that will run without SAR restriction are generally characterized by longer TR. As a result, a second consideration is the total number of image volumes that is achievable within the 7-hour scan time designated to dMRI protocols. The maximum 7-hour diffusion scan time is dictated by the overnight scan slot, accounting for the time required for sampling preparation, experiment setup, other imaging protocols, and scanner cleaning up. Given the total time available to the study in the scanner suite, it is ethical to acquire as much useful data as possible, resulting in our 7-hour target. Figure 1c demonstrates this dependence, which leads to the exclusion of all MB = 1 protocols as they cannot cover the needed set of diffusion directions (301 image volumes) in 7 hours.

The result of these evaluations are that (i) MB>1 is needed to achieve our desired q-space sampling and (ii) MB>2 would require sub-optimal TR to avoid SAR restrictions.

#### 2.1.3 Acceleration and image fidelity

Acceleration decisions also impact on image artifacts. In-plane acceleration along the phase encoding direction reduces blurring and distortion by reducing the effective echo spacing, while both slice-wise and in-plane acceleration lead to residual aliasing artifacts and noise amplification. Readout-segmented EPI reduces blurring and distortion at a cost of scan time, while acceleration along the phase encoding direction incurs a factor of $\sqrt{R}$ SNR loss for an R-fold acceleration. We explored the achievable parameter space of both segmentation and in-plane acceleration to determine the impact of different acceleration strategies on blurring, distortion, and residual aliasing artifacts.

**Fig. 2.**
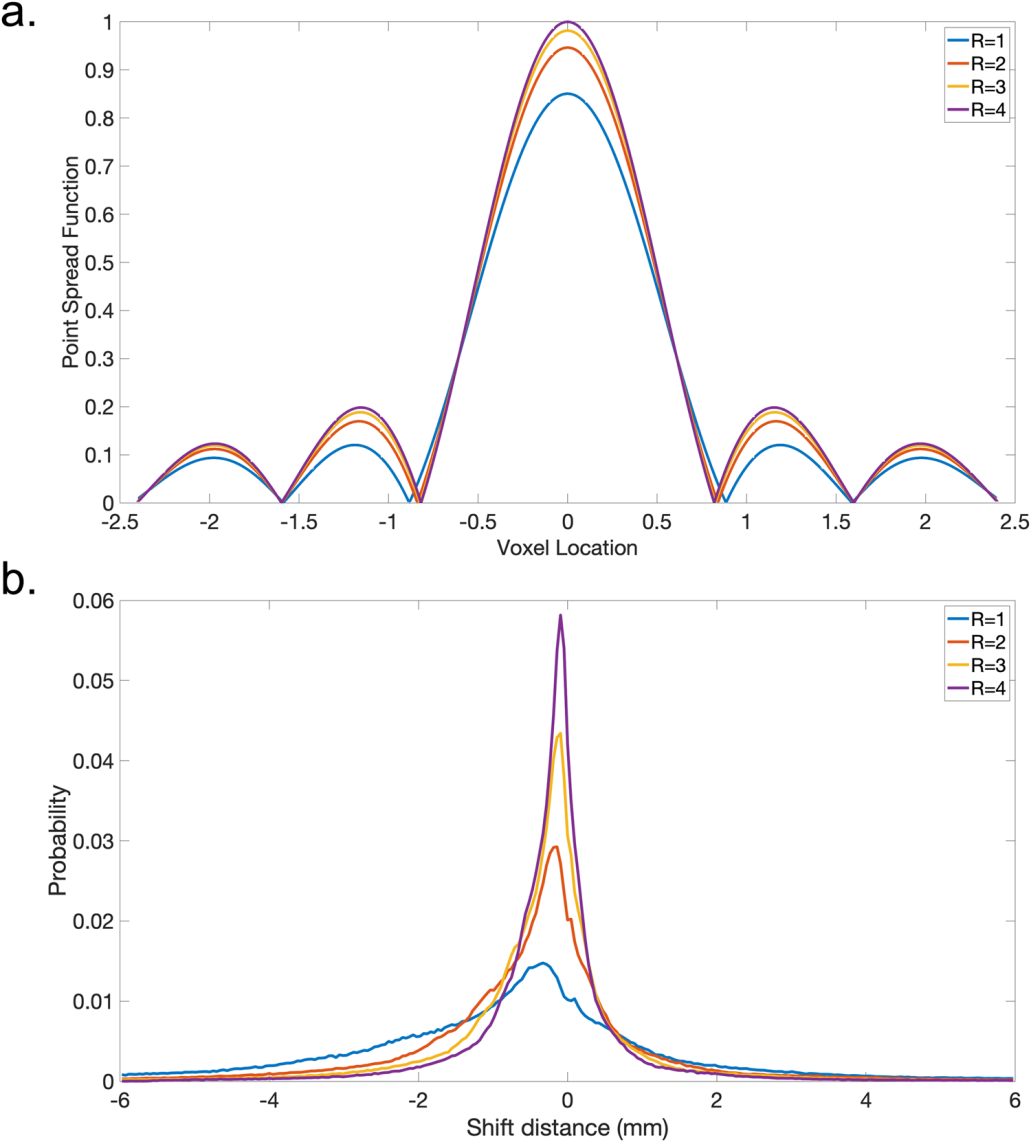
Simulation of blurring and distortion. (a) Blurring simulation: plot of voxel point spread functions along phase encoding direction for different phase encoding acceleration factors with an estimated T2* of 83 ms. (b) Distortion simulation: histogram of voxel shift distances (mm) simulated based on a representative B0 field map that was acquired at 3 T from a healthy infant and linearly scaled to 7 T.

Figure 2 shows a realistic simulation of blurring and distortion effects for different in-plane acceleration factors based on (i) an estimate of white matter T2* (83 ms) for the post-mortem dHCP infant and (ii) a representative B0 field map that was acquired at 3 T from a healthy infant and linearly extrapolated to 7 T. Blurring was measured using the full width at half maximum (FWHM) of the point spread function (Fig. 2a). Distortion was assessed with the 90th percentile of the shift distance of all voxels in the brain (Fig. 2b). Based on these simulations, R = 1 is expected to contain substantial distortion, with many voxels shifting over 2 mm (i.e., a shift of more than 3 voxels in our final target resolution). Using a small acceleration factor is expected to substantially reduce this artifact, with the largest reduction from R = 1 to 2. For blurring artifacts according to the FWHM measure, using higher acceleration factors only provides marginal improvement (Table 1).

**Table 1.** Quantitative measurement of image blurring and distortion for different phase encoding acceleration factors.

Phase encode acceleration	FWHM (voxel)	90th percentile shift distance (mm)
R = 1	1.29	2.16
R = 2	1.24	1.08
R = 3	1.23	0.72
R = 4	1.22	0.54

Image blurring is evaluated with the full width at half maximum (FWHM). Distortion is assessed with the 90th percentile of the shift distance of all voxels in the brain.

**Fig. 3.**
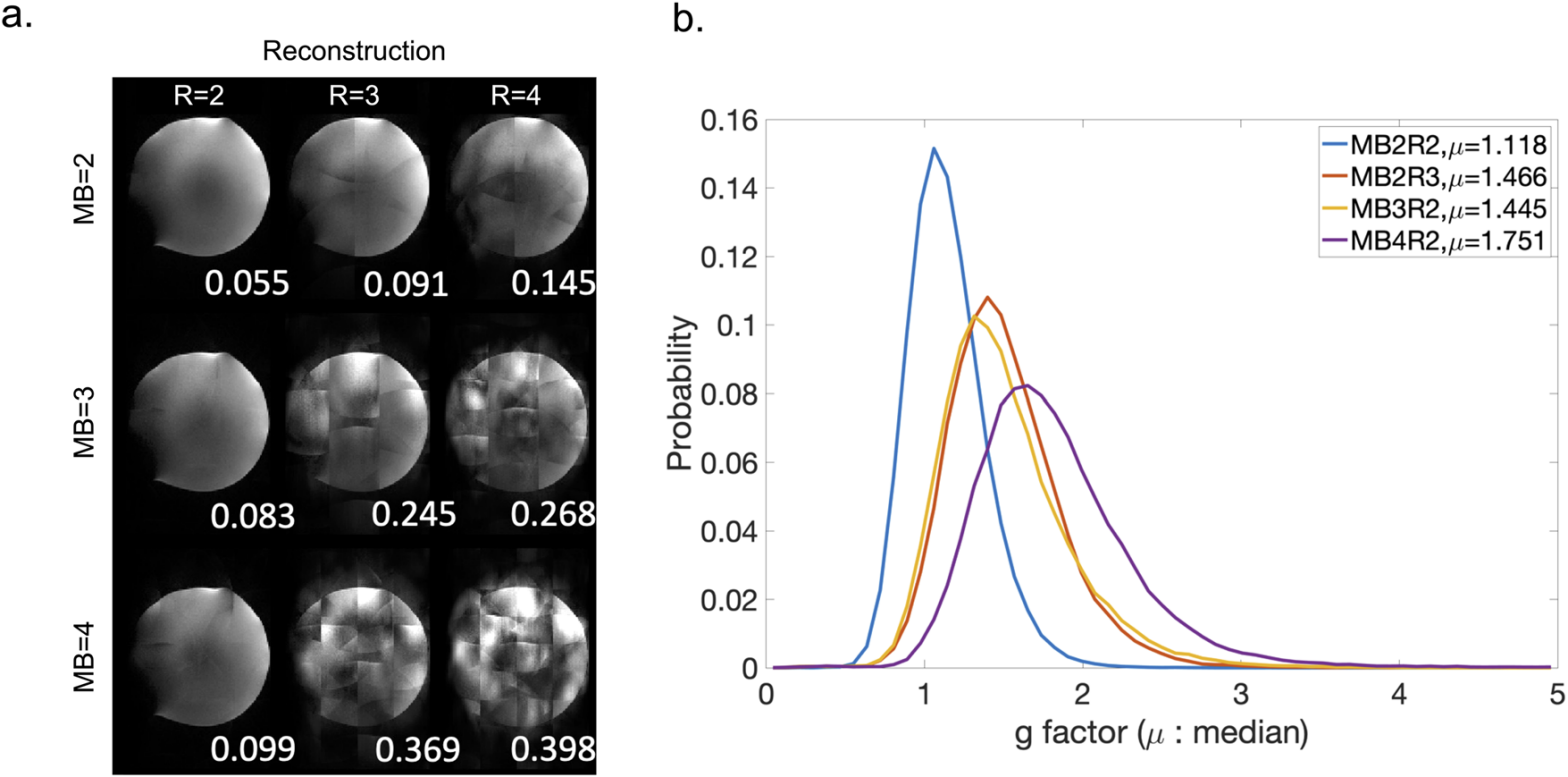
Evaluation of reconstruction performance for different acceleration protocols. (a) Reconstructed phantom images for different combinations of MB acceleration factors and phase encoding acceleration factors R. Normalized root mean square error is shown on the bottom right corner of each image. (b) Histogram of g-factors over all voxels for MB2R2, MB3R2, MB4R2, and MB2R3. µ = median g-factor.

We tested all protocols on a phantom and evaluated the quality of the reconstructed images. Figure 3 shows results with different combinations of MB = 2, 3, and 4 and R = 2, 3, and 4. The normalized root mean square error (NRMSE) was calculated for each result using MB = 1 image as a reference, which is shown on the bottom right corner of each image in Figure 3a. As expected, image quality is better with a lower acceleration factor, and reconstruction breaks down when both simultaneous multi-slice acceleration and phase encoding acceleration factors are high. The MB2R2 (i.e., MB factor 2 with in-plane phase encoding acceleration R = 2) result has the lowest NRMSE, followed by MB3R2, MB2R3, and MB4R2 results. These results suggest that, while the product of the two acceleration factors is generally the overriding factor, in practice the reconstruction tolerates high MB factors better than high in-plane acceleration factors.

These four protocols are further compared based on g-factor, which quantifies the reduction of SNR due to coil-geometry and sampling trajectory. Figure 3b shows the histogram of g-factor over all voxels for these four protocols, where MB2R2 protocol outperforms the other three by a large margin and, therefore, incurs a much lower SNR penalty. The overall SNR efficiency (SNR per unit time) of MB2R2 protocol is ~10% higher than the MB3R2 protocol, which is the second-best protocol.

The results of these evaluations of acceleration with respect to image quality are that (i) R>1 will provide major improvements in blurring and distortion and (ii) R>2 incurs major image artifacts. Hence, for in-plane accelerations R = 2 is the most robust option. Taking this together with the considerations from Section 2.1.2 regarding MB factors, we made the decision to use MB = 2 and R = 2.

#### 2.1.4 Final protocols

A major aim of the post-mortem dHCP study is to achieve higher resolution than can be achieved in vivo. Our protocol optimization targeted the maximum resolution that could be achieved under constraints relating to (i) isotropic resolution, (ii) target scan time, (iii) achievable acceleration, and (iv) maximum tolerable distortion/blurring. In order to acquire 301 dMRI volumes (detailed in Section 2.1.2) within the allotted 7 hours, the scan time per diffusion volume cannot exceed 83 s. As described in Sections 2.1.2 and 2.1.3, the optimal acceleration factors are R = 2 (in-plane) and MB = 2 (slice-wise). Under these constraints, achieving 0.8 mm resolution requires 7 readout segments. Further increase of resolution necessitates more readout segment, which would lead to a scan time longer than 7 hours and, therefore, was not considered.

The optimized dMRI protocol used a 2D simultaneous multi-slice spin-echo sequence with readout-segmented echo-planar imaging and monopolar diffusion preparation (Frost et al., 2015; Porter & Heidemann, 2009). The FOV was set to 150 × 150 × 104 mm^3^ to accommodate the head of a 44-week gestation infant measuring on the 95th percentile (Bastiani et al., 2019). Matrix size 188 × 188 × 130, 0.8 mm isotropic resolution, GRAPPA acceleration = 2, multi-band acceleration = 2, TR/TE = 10.8 s/113 ms, 7 readout segments, and 0.4 ms echo spacing. In total, 280 diffusion weighed volumes were acquired for three b values 3000, 6000, and 9000 s/mm^2^ with unique diffusion encoding directions (64, 88, and 128 directions, respectively). 21 b = 0 volumes were also acquired, with 17 using the same phase encoding direction as the high b-value data, and 4 with reversed phase encoding direction, to allow correction of susceptibility-induced image distortion (Andersson et al., 2003; Smith et al., 2004). The total acquisition time for dMRI data is just under 7 hours. A comparison between post-mortem and in vivo dMRI protocols is shown in Table 2.

**Table 2.** dMRI acquisition protocol comparison.

	Post-mortem	In vivo
Diffusion preparation	dwSE	dwSE
Readout trajectory	2D segmented (x7) EPI	2D single-shot EPI
Acceleration	MB2 + GRAPPA2	MB4
Resolution (mm)	0.8 x 0.8 x 0.8	1.5 x 1.5 x 1.5
TR (ms)	10800	3800
TE (ms)	113	90
Number of diffusion volumes	301	300
b values (s/mm^2^)	0, 3000, 6000, 9000	0, 400, 1000, 2600
Number of volumes/directions	21, 64, 88, 128	20, 64, 88, 128
Acquisition time	6 h 50 min approx.	19 min approx.

dwSE = diffusion-weighted spin echo; TR = repetition time; TE = echo time; EPI = echo planar imaging; MB = multiband; GRAPPA = generalized autocalibrating partially parallel acquisitions.

Based on the protocol, we can predict signal levels for DW-SE and compare them to known protocols. The HCP (Human Connectome Project) 7 T diffusion protocol (Vu et al., 2015) for b = 2000 s/mm^2^ requires TE = 71 ms, which is applied to adult brain with ADC≈1 µm^2^/ms (i.e., a factor of ~5 higher than predicted for post-mortem dHCP infants). For post-mortem infant brain at 7 T, we used T2 value of 280 ms (WM) (Thayyil et al., 2012) measured at 1.5 T and a linear correction of field-dependent T2 changes (Deistung et al., 2008; Uludağ et al., 2009), which gave predicted T2 values of 178 ms (WM). Previous studies have reported tissue ADC values from unfixed post-mortem infants, finding ADCs of 0.2-0.3 µm^2^/ms. Based on the estimated T2 and previously reported ADC, the raw signal level of WM for the post-mortem dHCP is predicted to be 2.5-fold higher than HCP. Given the HCP 7 T protocol uses 1.05 mm isotropic resolution with single-shot acquisition and the post-mortem dHCP uses 0.8 mm isotropic resolution with seven segments, the SNR of the post-mortem dHCP data will be about ~3-fold higher than HCP 7 T data. As we expect a small recruitment number for the post-mortem dHCP study, data quality in individual subjects is critical, hence we aim to exceed rather than match the SNR compared to HCP.

Structural scans were also acquired, which were based on the adult HCP (Van Essen et al., 2012) rather than the in vivo dHCP since the latter protocols were specifically designed to mitigate motion artifacts in vivo. A 3D MPRAGE (magnetization prepared rapid gradient echo) sequence was used to acquire T1-weighted data using the following parameters: TR = 2440 ms, TE = 3.35 ms, inversion time 1500 ms, flip angle 9º, field of view 150 mm × 150 mm × 102 mm, matrix size 384 × 384 × 256, 0.4 mm isotropic resolution, bandwidth 330 Hz/pixel, GRAPPA(Griswold et al., 2002) factor 2, echo spacing 6.7 ms, 4 repetitions, total scan time of 34 minutes. A 3D SPACE (sampling perfection with application-optimized contrast using different angle evolutions) sequence (Mugler, 2014) was used to acquire T2-weighted data using the parameters: TR = 2300 ms, TE = 586 ms, field of view 150 mm × 150 mm × 96 mm, matrix size 384 × 384 × 240, voxel size 0.4 mm isotropic, bandwidth 250 Hz/pixel, GRAPPA factor 2, echo spacing 6.72 ms, 6 repetitions, total scan time 58 minutes.

Infants were scanned on a Siemens Magnetom 7 T MRI scanner with a custom-built transmit/receive infant head coil (RAPID Biomedical) for this study, which consists of 2 transmit channels and 16 receive channels. The coil has an inner dimension of 117 mm (minimum diameter) × 135 mm (maximum diameter) × 114 mm (length), which can fit most infants up to term gestation. In the event of scanning a larger infant, a Nova head coil with 32 receive channels and a single transmit channel can be used. In addition, a 7 T-compatible acrylic cradle was custom-designed to optimally position the infant’s body within the RF coil and ensure minimal handling of the infant’s body. The cradle size is 500 mm (length) × 180 mm (width), with a narrowed head support section (50 mm) that fits within the infant head coil. The height of the cradle is adjustable such that the infant’s head can be placed at the center of the coil regardless of the head size.

Ethical approval was obtained from the South Central Oxford B NHS Research Ethics Committee (REC reference: 19/SC/0154), and research was conducted in accordance with standards set by Good Clinical Practice guidelines and the Declaration of Helsinki.

dMRI images were reconstructed off-line in MATLAB (Mathworks, Natick, MA) from raw k space data. The split slice-GRAPPA algorithm (Cauley et al., 2014) was used to separate aliased slices, followed by GRAPPA reconstruction of segmented EPI data with similar pipeline used previously (Frost et al., 2015). This large volume of raw data (~300 G) was incompatible with the reconstruction computer’s native storage, requiring data to be streamed from the scanner to an offline computer using the *iceNIH_RawSend* tool developed by Jacco A. de Zwart.

### 2.2 Cooling system

Lack of autoregulation in post-mortem tissue increases the risk of tissue heating. Substantial temperature rise during a scan has detrimental consequences to tissue, such as active damage or acceleration of degeneration (e.g., through autolysis). Additionally, temporally varying water diffusivity and tissue relaxation properties would affect quantitative measurements. Due to the sensitive nature of studies involving deceased neonates, we conservatively guarded against tissue damage through active cooling.

Tissue heating in MRI can occur due to two effects: (i) passive heating to match ambient temperature and (ii) active heating due to RF energy deposition. Neonates in our study arrive under mortuary conditions (9–13 °C) that are significantly colder than the ambient temperature (21°C) in the scanner room. Moreover, the use of dMRI protocols with high b value over long scan times generally increase ambient temperature due to heavy gradient duty cycle. In addition, the post-mortem dHCP scan will have significant energy deposition due to (a) high RF energy deposition at 7 T; (b) the spin-echo dMRI sequence with simultaneous multi-slice acquisition that involves intrinsically high energy deposition; and (c) heat accumulation over the long scan duration.

**Fig. 4.**
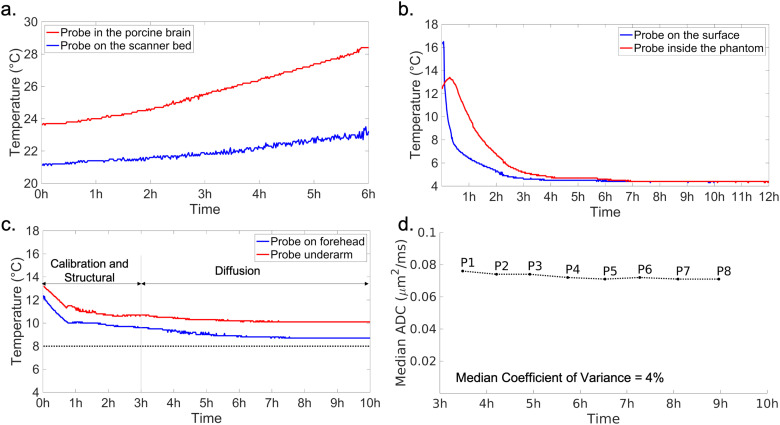
(a) Temperature of brain tissue (red) and ambient (blue) during a 6-hour diffusion scan with a porcine brain without active cooling. Active cooling was applied during data acquisitions for (b) and (c). (b) Temperature measured from the inside (red) and surface (blue) of a tissue-mimicking phantom. (c) Temperature measured during a post-mortem dHCP scan, with two probes placed on the forehead (blue) and underarm (red) of the infants. The target temperature 8 °C set for the cooler in the post-mortem dHCP scan is shown as a dotted horizontal line. (d) Median ADC of brain tissues calculated from the eight sub-groups of b = 9000 s/mm^2^ post-mortem dHCP data.

To investigate effects of both active and passive heating, we scanned an unfixed porcine brain using a candidate diffusion protocol with the highest b value of 4000 s/mm^2^ and measured tissue temperature continuously during the scan. The brain was extracted on the day of sacrifice and packed in a cylinder filled with fluorinert solution. The temperature was monitored using two fiber-optic temperature probes (Neoptix). One temperature probe was inserted into the brain to reflect the joint effects of active and passive heating and the other was placed at the end of scanner table to measure the ambient temperature. During a 6-hour diffusion scan, the tissue temperature measured inside the brain increased by 5°C, while the ambient temperature increased by 2°C (Fig. 4a). This would be consistent with both ambient temperature rises due to gradient coil heating (measured by the external probe) and additional heating due to RF energy deposition (reflected in the additional temperature rise in the internal probe). Notably, in this experiment there is no additional temperature variation due to mismatch between sample and ambient temperature, which would be the case in our scans of neonates that have been transferred from mortuary conditions to the scan room.

This experiment led to the development of a cooling system that not only aims to keep infant temperatures low to avoid tissue damage, but also to stabilize temperatures to avoid unwanted variation in MR signal properties during scanning. This work has been reported in a recent publication, focusing on the impact of temperature changes on the accuracy of quantitative tissue parameter measurements (including T1, T2, and diffusion parameters), and the benefit of using the developed cooling system (Rieger et al., 2023). During the scan, the infants were wrapped in three layers. The innermost layer was gauze, which was used to protect the skin from contact with the cooling blanket. The middle layer was the cooling blanket. The outer layer was cotton cloth used to block airflow, which might cause condensation on the surface of the cooling blanket. The temperature of the cooler was set to 8 °C. It was not considered important to set this temperature the same as the infant’s temperature at the start of the scanning (i.e., during calibration and structural scans), so there will be, in general, an initial settling period for the tissue temperature to stabilize.

### 2.3 Post-processing and modelling

dMRI data were pre-processed using the *top-up* and *eddy* tools (Andersson & Sotiropoulos, 2016) to correct for susceptibility and eddy current induced distortions. We calculate a transformation from diffusion space to the age-matched template (Serag et al., 2012) to perform automated tractography. *FLIRT* (Jenkinson & Smith, 2001) was used to register the average b = 9000 s/mm^2^ image to the T2-weighted structural with 6 degrees of freedom. A non-linear registration from T2-weighted image to the age-matched template was also performed with ANTs SyN (Avants et al., 2008). The linear transformation matrix was then combined with the non-linear field to produce the transformation needed in the tractography pipeline. Data analysis is presented for one infant, as the other infant had significant gross pathology, for which the current analysis pipeline has not been optimized.

Tissue masks for white matter, cortical gray matter, deep gray matter, and CSF was extracted from the structural T1w and T2w images based on the dHCP infant structural processing pipeline (Makropoulos et al., 2018) with modifications to address the strong bias field at 7 T. Specifically, the T2w/T1w ratio image was used as input instead of T2w image to eliminate receive field bias, and the FSL tool fsl_anat was subsequently used with the strong bias option to correct the transmit field bias.

After pre-processing, we analyzed the dMRI data using a combination of approaches to extract microstructurally relevant properties.

#### 2.3.1 Diffusion kurtosis model

The diffusion kurtosis imaging (DKI) model (Jensen et al., 2005) fits data at multiple b values to a second-order expansion to capture deviation from a Gaussian displacement distribution that reflects the degrees of tissue heterogeneity. DKI model parameters include the conventional diffusion tensor metrics (Basser et al., 1994) reflecting Gaussian behavior. We derived fractional anisotropy, mean diffusivity, principal diffusion direction, and mean kurtosis. The DKI model was fit to the data using FSL’s *dtifit* command.

#### 2.3.2 NODDI model

Data were also fit with the neurite orientation dispersion and density imaging (NODDI) model (Zhang et al., 2012). NODDI is a biophysically motivated model that attributes the dMRI signal to diffusion that is restricted (interpreted as “intra-neurite” spaces), hindered (“extra-neurite”) and unrestricted isotropic (CSF). The model also estimates an “orientation dispersion” index to account for fanning or crossing of neurite populations. In addition, an isotropic restricted compartment was included, which is common for post-mortem tissue (Alexander et al., 2010). NODDI was fit using the GPU-based *cudimot* tool, which reduces the otherwise long computational times for NODDI fitting (Hernández et al., 2013).

We examined a number of axial diffusivities for NODDI fitting from 0.01 µm^2^/ms to 1 µm^2^/ms with an interval of 0.05 µm^2^/ms. We used the Bayesian Information Criterion, BIC, to evaluate the fitting performance, which is a standard model selection criterion that quantifies the quality of data fitting while accounting for model complexity. For fast computation, four axial slices at lateral ventricle position were used for the optimization.

The averaged BIC value was calculated for gray and white matter using a brain mask excluding background and CSF, which was used as a selection criterion for the optimal axial diffusivity value. Among all axial diffusivities assessed, 0.3 µm^2^/ms provides the minimum BIC value (Supplementary Fig. S1a), which was then used for our final NODDI fitting.

#### 2.3.3 Constrained spherical deconvolution

Constrained spherical deconvolution (CSD) analysis (Tournier et al., 2007) allows depiction of more complex fiber structures. CSD analyses were performed with MRtrix3 (Tournier et al., 2012) using multi-shell multi-tissue CSD algorithm, which estimates tissue-specific fiber orientation distribution function based on different b-value dependencies of different tissue types. Three tissue response functions were estimated from the data using *dwi2response* function (Dhollander, 2016, 2019). These responses functions were then used in orientation distribution function fitting. Bias field correction was performed with *mtnormalise* function.

#### 2.3.4 Tractography

Finally, diffusion data were fit using a ball-and-stick model (Behrens et al., 2007; Jbabdi et al., 2012) that estimates fiber orientation for multiple fiber populations from each voxel. Tractography was then performed with the infant-specific “baby autoPtx” tractography pipeline developed for dHCP dMRI processing (Bastiani et al., 2019). This pipeline dissects 16 white matter tracts, including projection, association, callosal, cerebellar, and limbic fibers. These segmented tract masks were used for tract-specific microstructure analysis.

## 3 Results

### 3.1 Effects of active cooling

As shown in Figure 4c, when using the developed cooling system, the temperature measured from the underarm decreases from 13.2 °C to 10.7 °C in the first 3 hours, which are calibration, structural, and pilot scans quantitative multi-parameter mapping (see Section 4 for details), and then slowly decreases by no more than 1 °C over the following 7-hour diffusion scan with a final temperature of 10.1 °C. The temperature at the forehead decreases from 12.4 °C to 9.6 °C during the first 3 hours and decreases by no more than 1 °C over the following 7 hours ending at 8.7 °C.

To evaluate the stability of tissue ADC during the dMRI scan, ADC values were calculated for each of the eight b = 9000 s/mm^2^ sub-groups. These sub-group data were acquired at times relatively evenly distributed across the 7-hour dMRI acquisition, allowing us to assess the variation of ADC during the scan. Figure 4d shows the plot of median ADC for white matter and gray matter, which starts at 0.076 µm^2^/ms and gradually converges to a stable value around 0.071 µm^2^/ms. The median coefficient of covariance for white matter and gray matter is 4%, indicating high stability. The results demonstrate the active cooling system is able to maintain a stable temperature in the post-mortem tissue and thus stable ADC values during the dMRI scan. Although it might have been preferable to monitor the ADC changes using the lower b-value measurements since non-Gaussianities would be less prominent, the b = 9000 s/mm^2^ data enabled us to track the entire experiment.

### 3.2 dMRI results

**Fig. 5.**
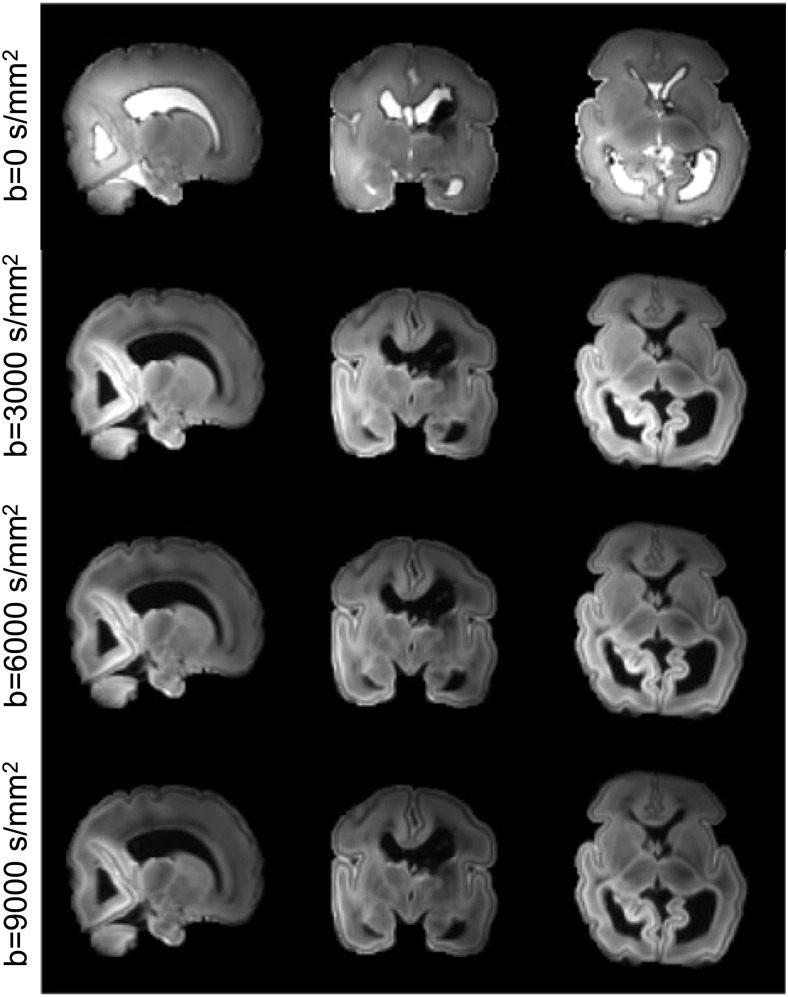
Post-mortem dHCP images from one of the first two subjects. Three b shells (3000, 6000, and 9000 s/mm^2^) were acquired at 0.8 mm isotropic resolution. Here the b = 0 images and shell-averaged diffusion weighted images are shown. Note this infant had bilateral grade 3 intraventricular hemorrhages (IVH) and post-hemorrhagic ventricular dilatation, as can be observed from the images.

Figure 5 shows multi-shell dMRI images acquired from the post-mortem dHCP subject. The high spatial resolution of the data allows delineation of detailed structures, such as the cortical plate, which is only 1–2 voxels thick even at this high resolution. Blurring and distortion is negligible due to the use of readout-segmented EPI and in-plane parallel acceleration. Despite the small voxel size, the data have high SNR. Due to the nature of this study, subjects will be expected to have a range of pathologies. This infant had bilateral grade 3 intraventricular hemorrhages (IVH) and post-hemorrhagic ventricular dilatation, as can be observed from the images.

**Fig. 6.**
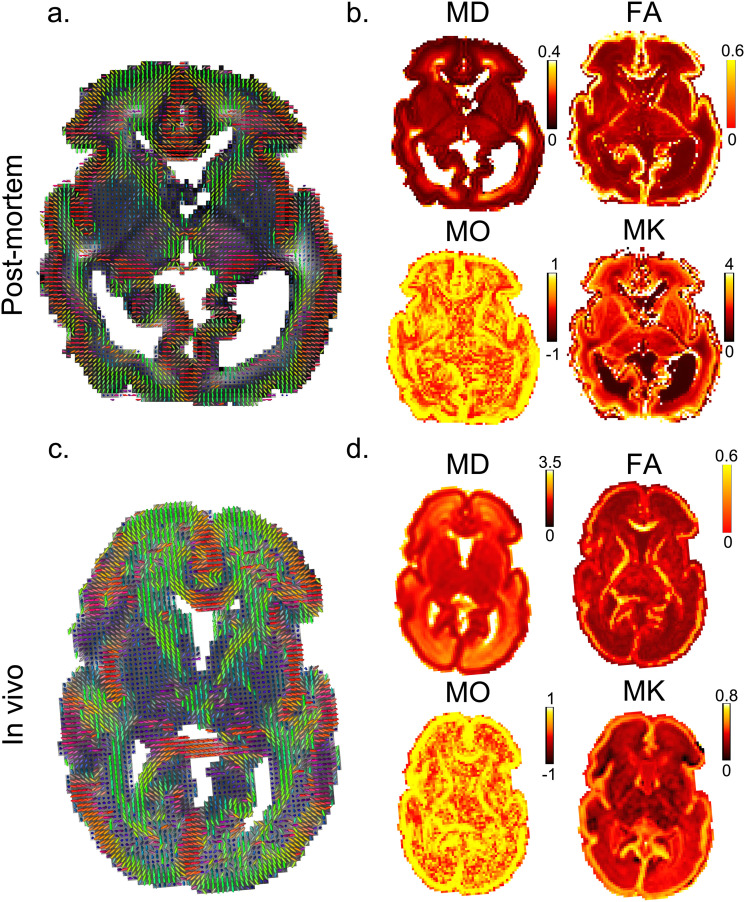
Assessment of diffusion kurtosis model fit. There is a high degree of qualitative correspondence in spatial patterns between the post-mortem data and the exemplar age-matched in vivo subject. In general, greater microstructural detail can be resolved in the post-mortem data relative to the in vivo data due to the higher spatial resolution. Note the acquisition time for the post-mortem data is much longer than the in vivo data (7 hours vs 19 minutes). Principal diffusion direction from DKI model fit of post-mortem (a) and in vivo (c) dHCP data. FA (fractional anisotropy), MO (mode of the anisotropy), MD (Mean diffusivity, expressed in µm^2^/ms), and MK (mean kurtosis) parameters from the DKI model fit of post-mortem (b) and in vivo (d) dHCP data. Quantitatively, overall spatial patterns of DKI parameters are consistent between subjects. However, in the post-mortem data, the MD values are noticeably lower, while the MK values are noticeably higher, than the in vivo data. Note the change of color bar scale between the post-mortem and in vivo maps. The post-mortem data has a resolution of 0.8 mm isotropic, and the in vivo data have a resolution of 1.5 mm isotropic (but were interpolated to 1.17 x 1.17 x 1.5 mm^3^ in post-processing).

**Fig. 7.**
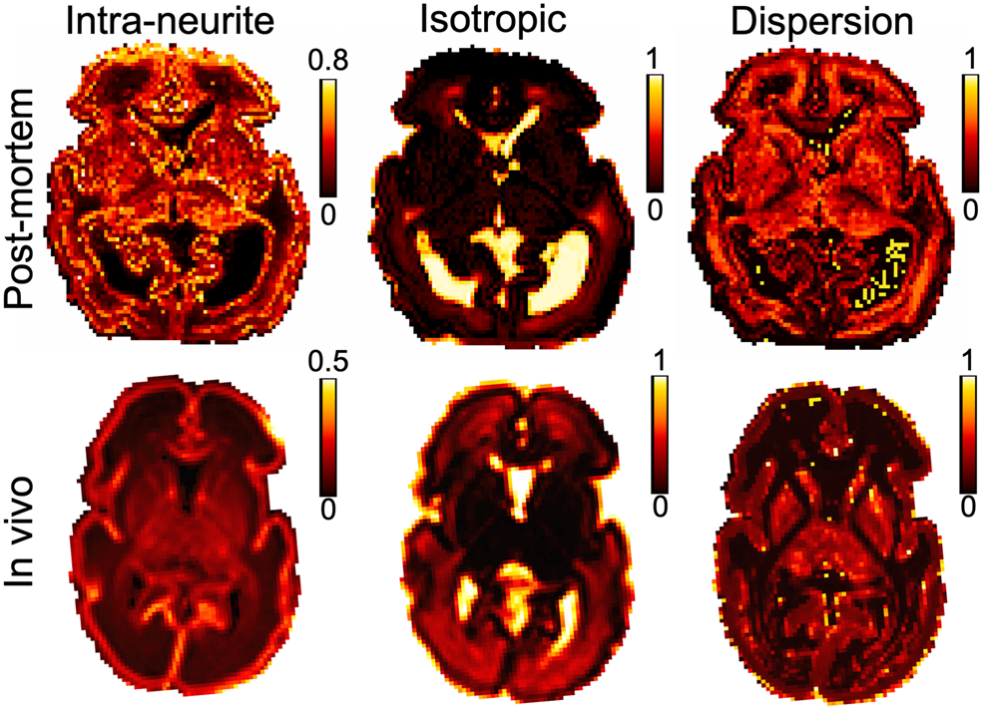
Assessment of NODDI model fit results. The parameter maps are “Intra-neurite volume fraction”, “isotropic volume fraction”, and “orientation dispersion” index parameters from NODDI model fit of post-mortem and the exemplar age-matched in vivo subject. Overall, the spatial patterns of NODDI parameters are quantitatively consistent between subjects. However, in the post-mortem data, the “intra-neurite volume fraction” values are noticeably higher than the in vivo data. Note the change of color bar scale between the post-mortem and in vivo maps. The post-mortem data have a resolution of 0.8 mm isotropic, and the in vivo data have a resolution of 1.5 mm isotropic (but were interpolated to 1.17 x 1.17 x 1.5 mm^3^ in post-processing).

We compared the dMRI data acquired post-mortem to an age-matched in vivo subject. Despite the 6.6-fold smaller voxel volume of post-mortem images (0.512 vs 3.375 mm^3^), there was sufficient SNR to support robust diffusion model fitting, including DKI (Fig. 6) and NODDI (Fig. 7). These datasets demonstrate consistency in overall spatial patterns of model parameters between the post-mortem and in vivo data, as well as notable differences. Compared to the in vivo data, the post-mortem data exhibited higher mean kurtosis (MK) and higher “intra-neurite volume fraction” from the NODDI model (note the differences in color bar scale).

Additionally, the post-mortem mean diffusivity (MD) is considerably lower than that of the in vivo data, consistent with previous post-mortem studies in both fixed and unfixed tissue (D’Arceuil & de Crespigny, 2007; Miller et al., 2011). Lower post-mortem body temperature (10 °C vs 37 °C) is predicted to cause an ~2-fold reduction in water diffusivity compared to in vivo, which is broadly consistent with the reduction of MD in CSF by a factor of 2.8. Notably, a much larger difference in MD is observed in brain tissue (~10-fold lower in the post-mortem). These differences might be explained by post-mortem changes such as cell swelling (Toorn et al., 1996; Xiao et al., 2020) and the higher b values used for the post-mortem scans, which is more sensitive to the slow-diffusion component and would lead to reduced MD. Shell-wise calculation of MD and attenuation factor for white matter, gray matter, and CSF is summarized in Supplementary Table 1.

Caution is required in interpreting NODDI results due to established model degeneracies for specific parameters (Jelescu et al., 2016). In particular, we found high variability in the “dispersion” parameter depending on the (fixed) value of the axial diffusivity used in the fitting (Supplementary Fig. S1b). The BIC curve is relatively flat near the minimum (i.e., for axial diffusivities between 0.15 and 0.45 µm^2^/ms), but the mean orientation dispersion presents a large variation. For example, in the NODDI fitting, the mean orientation dispersion presents over 20% changes between the optimal axial diffusivity (0.3 µm^2^/ms, selected based on BIC criterion) and the two nearby axial diffusivities assessed (i.e., 0.25 and 0.35 µm^2^/ms). Inaccuracies in diffusivity can lead to biased “dispersion” estimation in NODDI fitting (Howard et al., 2022; Yi et al., 2019).

**Fig. 8.**
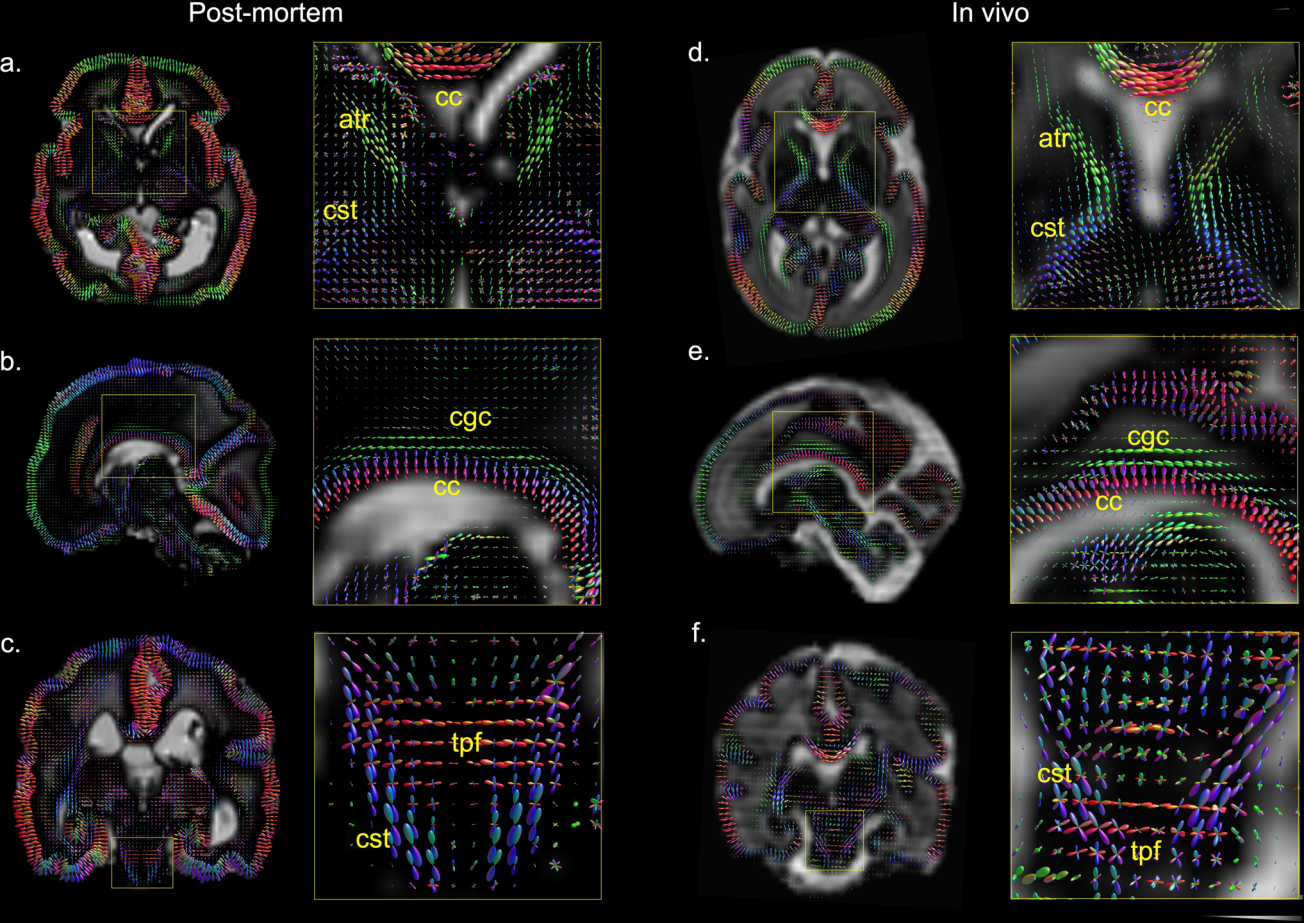
Axial (a, d), sagittal (b, e) and coronal (c, f) sections of white matter ODFs overlaid on CSF components (background) for post-mortem dHCP data and data from the age-matched in vivo dHCP subject. Abbreviations: cc = corpus callosum; atr = anterior thalamic radiation; cst = corticospinal tract; cgc = cingulate gyrus part of the cingulum; tpf = transverse pontine fibers.

White matter orientation distribution functions (ODFs) from CSD analyses of the post-mortem data and the data from the age-matched in vivo subject demonstrate high similarity (Fig. 8). Microstructural organization of the cortical plate is dominantly radial, as reflected by the ODFs being radial with respect to the cortical space, which is consistent with histological studies (Marín‐Padilla, 1992) and diffusion MRI (McKinstry, 2002) conducted at this gestational age. Early emerging white matter fiber pathways are observed in both datasets, such as corpus callosum (cc), anterior thalamic radiation (atr), cingulate gyrus part of the cingulum (cgc), and corticospinal tracts (cst). The coronal detail views of the brainstems (Fig. 8c,f) show the crossing fibers in the pons between cst (blue) that runs superior-inferior and the transverse pontine fibers (tpf, red) that runs left–right. These crossing fibers are more clearly depicted in the post-mortem data, which might be due to the improved spatial resolution and SNR.

**Fig. 9.**
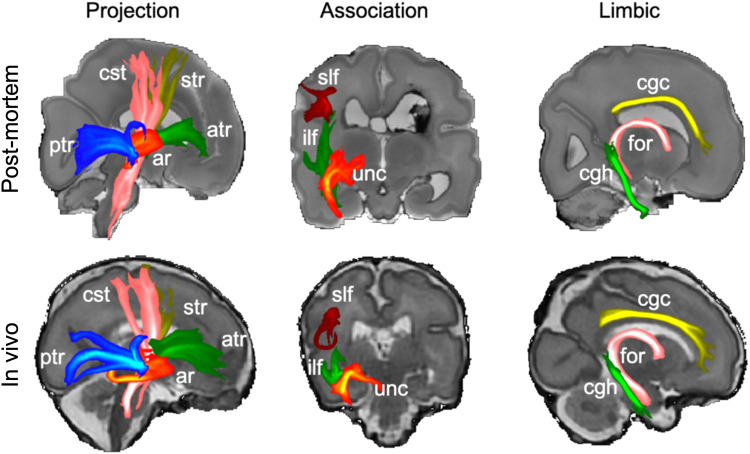
Maximum intensity projections of 11 tracts from the in vivo and post-mortem data. All tracts are visualized using the same thresholds (0.005, 0.05). Abbreviations: ar = acoustic radiation; atr = anterior thalamic radiation; cgc = cingulate gyrus part of the cingulum; cgh = parahippocampal part of the cingulum; cst = corticospinal tract; for = fornix; ilf = inferior longitudinal fasciculus; ptr = posterior thalamic radiation; slf = superior longitudinal fasciculus; str = superior thalamic radiation; unc = uncinate fasciculus.

Finally, we examine white matter tracts using probabilistic tractography and dHCP analysis protocols (Bastiani et al., 2019). We estimated 11 tracts in the right cerebral hemisphere (Fig. 9a) including five projection tracts (corticospinal tract, auditory radiations, and anterior, posterior, and superior thalamic radiations), three association tracts (uncinate, superior longitudinal, and inferior longitudinal fasciculus) and three limbic tracts (fornix, cingulate, and hippocampal cingulum bundle) (Bastiani et al., 2019; Ouyang et al., 2019). The result demonstrates the feasibility of performing probabilistic tractography on unfixed post-mortem data using the same analysis pipeline developed for in vivo dHCP data.

## 4 Discussions

The post-mortem dHCP study aims to acquire and openly distribute high-quality diffusion imaging of unfixed post-mortem infant brains that will provide insight into developing brain connectivity and microstructure. The protocols are designed to be as general as possible without focusing on specific output maps. To be consistent with in vivo dHCP, most diffusion acquisition parameters were matched to the in vivo dHCP dataset.

Imaging unfixed brains in situ provides several advantages over imaging fixed brains. A primary advantage is that imaging unfixed brains requires no brain donation. This creates a more acceptable conditions for study participation, which can facilitate the collection of sensitive imaging datasets. Due to ischemic changes and low temperature, diffusivity of unfixed post-mortem tissue is reduced compared to in vivo, but it is still higher than the diffusivity of fixed tissue measured at room temperature (~0.08 µm^2^/ms for brain white matter measured with b = 4500 s/mm^2^) (Miller et al., 2011; Roebroeck et al., 2019). In addition, the T_2_ of unfixed tissue is significantly higher than fixed tissue. Together, they enable diffusion MRI of unfixed brain with higher resolution and SNR than that can be achieved in fixed brains.

A major aim of the post-mortem dHCP study is the development of a bespoke experimental setup and imaging protocols that are compatible with in vivo dHCP, while providing high image quality that are not achievable in vivo. This motivated our decisions on imaging sequence and acquisition parameters. The most common approach for in vivo diffusion MRI is to use single-shot EPI with in-plane acceleration. However, we did not consider single-shot EPI in the post-mortem study as it would suffer from significant distortion and T2* blurring for high-resolution scans, because of the long echo spacing (>1 ms on our scanner) and the large matrix size required for sub-millimeter resolution. Parallel acceleration along the EPI phase encoding direction could mitigate those artifacts, but to achieve similar level of distortion and blurring as the readout-segmented EPI sequence used in this study, single-shot EPI requires a high under-sampling factor of 5 (Supplementary Fig. 2 and Supplementary Table 2), which is not feasible with the 16-channel neonate coil. Therefore, we utilized readout-segmented EPI, which reduces distortion by breaking up the 3D volume into readout segments (Holdsworth et al., 2008; Porter & Heidemann, 2009). However, the entire 3D volume takes longer to acquire due to the increased number of readouts. To acquire our target number of diffusion volumes in an overnight scan, we needed to reduce the scan time per volume. This was achieved by incorporating accelerations through plane with multi-band excitation, which reduces the TR, and in plane with GRAPPA under-sampling, which reduces the number of readout segments needed. The in-plane and through-plane accelerations compete for the use of the same coil encoding information. High accelerations along both dimensions would lead to image reconstruction artifacts. The optimal combination of in-plane and through-plane acceleration depends on various factors, including the number of receive coils and their geometry, field strength, sample size, and readout trajectory. This dependency has not been investigated using the same experimental setup as developed in this study. Therefore, we decided to evaluate different combinations of GRAPPA and multi-band factors to find the best choice that can provide high data quality within the scan time limit. This resulted in the final decision of GRAPPA R = 2 and multi-band factor 2, which coincidentally matches to the acceleration parameters used in many in vivo diffusion studies. Partial Fourier is commonly used with single-shot EPI to achieve a shorter TE for better SNR, but it was not used in this study as it would introduce further blurring along the phase encoding direction. By integrating readout segmentation, multi-slice acceleration and in-plane acceleration (Frost et al., 2015), along with the use of 7 T and a bespoke 16-channel infant head coil, we were able to achieve high-quality dMRI data with the targeted spatial resolution, number of diffusion directions and b values, and total scan time.

Tissue heating proved to be a further major technical challenge. Previous work in post-mortem dMRI of fixed tissue has established that long scan times coupled with the need for high b value lead to significant gradient heating, which raises the ambient room temperature and can passively heat samples (Miller et al., 2011). Our study conditions introduced additional concerns regarding RF energy deposition at 7 T for post-mortem unfixed tissue, which lacks autoregulation and is susceptible to damage. Tissue integrity must be preserved for ethical reasons and to avoid compromise of later autopsy examination. To address this challenge, we used an in-house developed active cooling system, which maintains stable low body temperature during dMRI scanning. This system also improves the stability of tissue properties such as T1, T2, and diffusivities during scanning.

For the DKI model parameters, MD values are reduced globally in the post-mortem data, which is only partially accounted for by tissue temperature differences. Additionally, MK values are higher globally in the post-mortem data. Together these differences suggest greater microstructural complexity and more restricted water movement in the post-mortem data, which could potentially be due to cell swelling during the peri- and post-mortem period. Such changes are a direct consequence of the processes of cell death (D’Arcy, 2019) with the peak in intracellular volume expansion being delayed at reduced temperatures (Janaway et al., 2009). There are, however, many factors surrounding the physiological state of the infant prior to death that could compound and accelerate these cell changes, such as infection and resultant cytokine release, degree of supplemental oxygen, and use of intravenous fluids (McAdams & Juul, 2012; Truttmann et al., 2020). In addition, the higher b values used in the post-mortem scans led to an increased sensitivity to the slow-diffusion component, which might also contribute to the difference in MD between the in vivo and the post-mortem dHCP data.

Although the main focus of this study is to estimate diffusion parameters, other tissue parameters such as relaxation times can offer important complementary information, providing a comprehensive assessment of tissue structure. In the first two post-mortem scans, we piloted quantitative multi-parameter mapping sequences (Weiskopf et al., 2013), which can provide T1, T2*, proton density, and magnetic transfer saturation at 0.8 mm isotropic resolution in a ~1.5-hour scan. While these data were not usable due to image artifacts, we will amend these protocols and aim to include these sequences in future infants. In addition, we will also explore the feasibility of acquiring T2 maps, with the main challenges being the high sensitivity to B1+ field inhomogeneity and long scan time for high-resolution whole brain coverage.

In this study, the imaging protocols and scan setup were optimized under the limitation of scan time, hardware, and ethic requirement, while pursuing compatibility with the in vivo dHCP acquisition. However, under a different setup, there are certain things in this study that can be improved. For example, a higher multi-band acceleration may be possible using an RF receive coil with more receive channels, which could be used to achieve a higher spatial and angular resolution for diffusion MRI data using the same scan time. The low diffusivity of the post-mortem tissue and the b values used in this study may lead to reduced diffusion contrast compared to the in vivo dHCP data. If more powerful gradient coils are available, it is possible to further increase the b values to improve the diffusion contrast and the sensitivity to restricted diffusion, which could facilitate fitting with advanced diffusion models (Assaf et al., 2008).

The recruitment of this study was halted due to the COVID-19 pandemic. When recruitment resumes, we aim to investigate how representative these current results are of infants studied under these conditions. We believe future recruitment will be feasible based on the implementation of a tailored care pathway and bespoke scanning setup yielding high-quality data, which has been well received by parents, the clinical team, and the research imaging center. Our ultimate aim is for this open dataset to accelerate advances in our understanding of the developing human brain and the impact of pathology and death during the term and preterm periods.

## Supplementary Material

Supplementary Material

## Data Availability

Due to ethical restrictions, it is appropriate to monitor access and usage of the data as it include highly sensitive information. Data sharing requests should be directed to rebeccah.slater@paediatrics.ox.ac.uk and karla.miller@ndcn.ox.ac.uk. The dHCP data (Second Data Release) that used in this work are available online (http://www.developingconnectome.org/second-data-release). FSL tools for dMRI processing and analysis are available via FSL (https://fsl.fmrib.ox.ac.uk/fsl/fslwiki). FSL Eddy tool for dMRI data preprocessing are available via https://fsl.fmrib.ox.ac.uk/fsl/fslwiki/eddy. DKI fitting was performed with FSL’s dtifit tool (https://fsl.fmrib.ox.ac.uk/fsl/fslwiki/FDT). Bias field correction was performed with FSL’s *fsl_anat* tool (https://fsl.fmrib.ox.ac.uk/fsl/fslwiki/fsl_anat). Linear image registration was performed with FSL *FLIRT* (https://fsl.fmrib.ox.ac.uk/fsl/fslwiki/FLIRT). Cudimot was used for NODDI fitting (https://users.fmrib.ox.ac.uk/~moisesf/cudimot/index.html). Tractography was performed with Baby Autoptx tool developed for the dHCP (https://doi.org/10.1016/j.neuroimage.2018.05.064). CSD analyses were performed with MRtrix3 (https://www.mrtrix.org/). Processing pipeline for T2w structural data is available online (https://github.com/BioMedIA/dhcp-structural-pipeline).
