# Supplementary material for: High resolution diffusion imaging in the unfixed post-mortem infant brain at 7T
Source: Imaging Neurosci (Camb). Author manuscript; Available in PMC 2025 Jul 21. (PMC12043270; doi:10.1162/imag_a_00069)
Supplement: Supplementary Information [file EMS207277-supplement-Supplementary_Information.pdf]

Supplementary Information

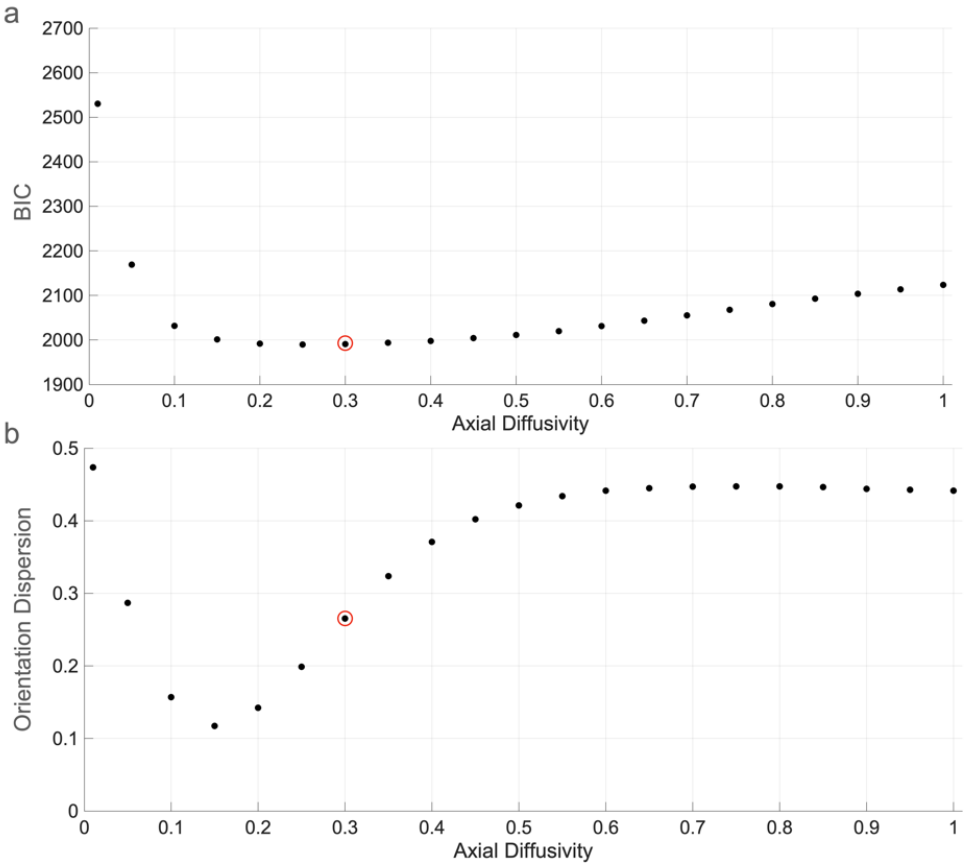

Supplementary Figure 1. Mean Bayesian Information Criterion (BIC) and orientation dispersion value of NODDI fitting with different axial diffusivities, calculated for gray and white matter using a brain mask excluding the background and CSF. For fast computation, 4 axial slices lateral ventricle position were used for the optimization. Among all axial diffusivities assessed, 0.3 $\mu m^2/ms$ provides the minimum BIC value (indicated by the red circle in Fig. 1a), which was then used for our final NODDI fitting. The mean orientation dispersion changes significantly across different axial diffusivities, with a minimum/maximum value of 0.117/0.474. The BIC curve is relatively flat near the minimum (i.e., for axial diffusivities between 0.15 and 0.45 $\mu m^2/ms$), but the mean orientation dispersion presents a large variation. For example, in the vicinity of the optimal axial diffusivity (i.e., 0.3 $\mu m^2/ms$), the mean orientation dispersion presents over 20% changes between the optimal axial diffusivity and the two nearby axial diffusivities assessed (i.e., 0.25 and 0.35 $\mu m^2/ms$).

	White matter (MD/Attenuation)	Cortical gray matter (MD/Attenuation)	Deep Gray matter (MD/Attenuation)	CSF (MD/Attenuation)
b=3000 mm^2/s	0.159/62.1%	0.099/74.3%	0.125/68.7%	1.034/4.5%
b=6000 mm^2/s	0.116/49.9%	0.081/61.5%	0.094/56.9%	0.535/4.04%
b=9000 mm^2/s	0.093/43.3%	0.071/52.8%	0.079/49.1%	0.36/3.92%

Supplementary Table 1. Averaged mean diffusivity (MD) and attenuation (calculated as exp(-MD*b)) for white matter, cortical gray matter, deep gray matter, and CSF. The unit of MD is $\mu m^2/ms$.

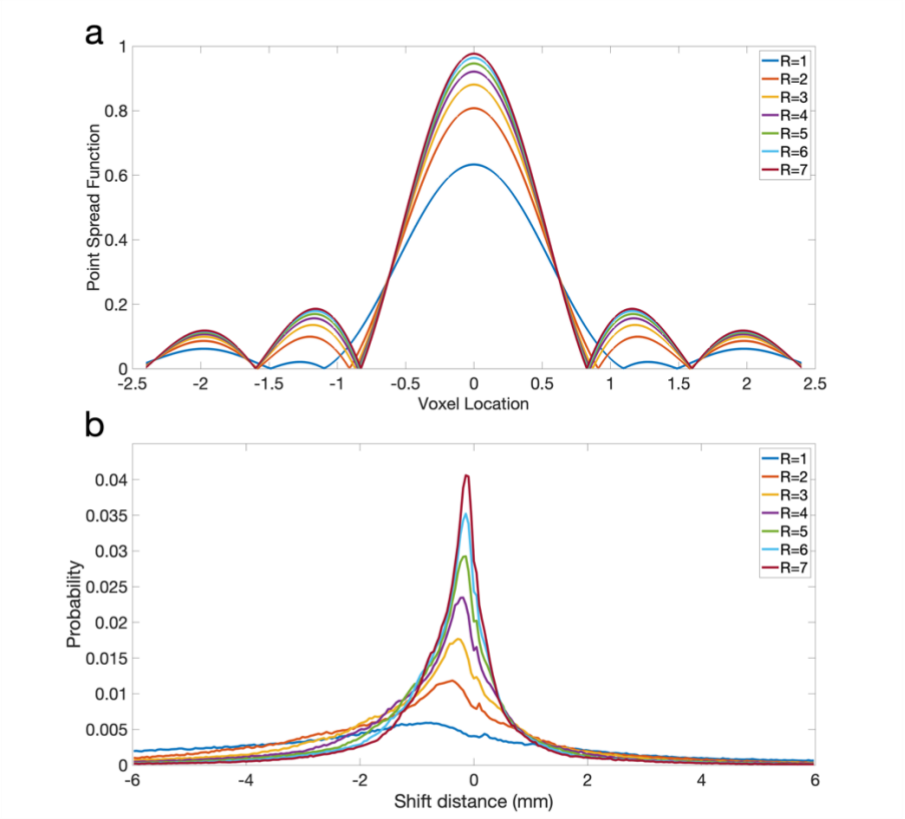

Supplementary Figure. 2. Simulation of blurring and distortion with a single-shot EPI acquisition. a) Blurring simulation: plot of voxel point spread functions along phase encoding direction for different phase encoding acceleration factors with an estimated T2* of 83ms. b) Distortion simulation: histogram of voxel shift distances (mm) simulated based on a representative B0 field map that was acquired at 3T from a healthy infant and linearly scaled to 7T. Seven in-plane acceleration factors are assessed. The echo spacing is 1ms in the simulation. To achieve the same level of distortion and blurring as the readout-segmented EPI sequence used in this study, single-shot EPI should be acquired with R=5 to match the effective echo spacing.

**Phase encode acceleration**	**FWHM (voxel)**	**90th percentile shift distance (mm)**
R=1	1.44	5.40
R=2	1.31	2.70
R=3	1.27	1.80
R=4	1.25	1.35
R=5	1.24	1.08
R=6	1.24	0.90
R=7	1.23	0.77

Supplementary Table 2. Quantitative measurement of image blurring and distortion for different phase encoding acceleration factors using single-shot EPI readout. Image blurring is evaluated with the full width at half maximum (FWHM). Distortion is assessed with the 90th percentile of the shift distance of all voxels in the brain. Seven in-plane acceleration factors are assessed. The echo spacing is 1ms in the simulation. To achieve the same level of distortion and blurring as the readout-segmented EPI sequence used in this study, single-shot EPI should be acquired with R=5 to match the effective echo spacing.
